# Oncogenic functions of IGF1R and INSR in prostate cancer include enhanced tumor growth, cell migration and angiogenesis

**DOI:** 10.18632/oncotarget.1884

**Published:** 2014-04-02

**Authors:** Isabel Heidegger, Johann Kern, Philipp Ofer, Helmut Klocker, Petra Massoner

**Affiliations:** ^1^ Division of Experimental Urology, Department of Urology, Innsbruck Medical University, Innsbruck, Austria; ^2^ Department of Hematology and Oncology, Innsbruck Medical University, Innsbruck, Austria

**Keywords:** Prostate cancer, IGF targeting therapies, insulin-like growth factor receptor-1 (IGF1R), insulin receptor (INSR)

## Abstract

We scrutinized the effect of insulin receptor (INSR) in addition to IGF1R in PCa using *in vitro* and *in vivo* models. *In-vitro* overexpression of IGF1R and INSRA, but not INSRB increased cell proliferation, colony formation, migration, invasion and resistance to apoptosis in prostate cancer cells (DU145, LNCaP, PC3). Opposite effects were induced by downregulation of IGF1R and total INSR, but not INSRB. In contrast to tumor cells, non-cancerous epithelial cells of the prostate (EP156T, RWPE-1) were inhibited on overexpression and stimulated by knockdown of receptors. *In-vivo* analyses using the chicken allantoic membrane assay confirmed the tumorigenic effects of IGF1R and INSR. Apart from promoting tumor growth, IGF1R and INSR overexpression also enhanced angiogenesis indicated by higher vessel density and increased number of desmin-immunoreactive pericytes. Our study underscores the oncogenic impact of IGF1R including significant effects on tumor growth, cell migration, sensitivity to apoptotic/chemotherapeutic agents and angiogenesis, and characterizes the INSR, in particular the isoform INSRA, as additional cancer-promoting receptor in prostate cancer. Both, the insulin-like growth factor receptor 1 and the insulin receptor exert oncogenic functions, thus proposing that both receptors need to be considered in therapeutic settings.

## INTRODUCTION

Prostate cancer (PCa) is the leading cancer entity in men [1]. While localized PCa is potentially curable by surgery or by radiation therapy, metastatic PCa has a high likelihood to progress to a fatal disease stage. Many efforts employing targeting approaches focus on the development of new therapeutic options for the treatment of advanced stages of PCa. Some new molecular targeting drugs like Abiraterone acetate (Zytiga®) blocking a key enzyme of androgen biogenesis or MDV 3100 (Enzalutamide®) inhibiting the androgen receptor were recently approved and successfully entered clinical routine of metastatic PCa treatment. Other molecular targeting drugs like anti-angiogenic agents, novel anti-androgens and modulators of the immune response or the bone environment of metastases are under investigation in clinical studies [2,3,4].

A pivotal regulatory network and attractive therapeutic target in oncology is the insulin-like growth factor (IGF) axis. Several neutralizing antibodies or small molecule receptor kinase inhibitors have been developed for targeting the IGF1 receptors (IGF1R) and are tested in clinical studies in various cancer entities. Enhanced stimulation of the IGF network has been associated with carcinogenesis and tumor progression in several tumor types including PCa ([5,6] and references therein). Numerous studies have been performed to correlate serum levels of IGF-1 and IGF-binding protein-3 (IGFBP3) with the risk for PCa. Although results were heterogeneous meta-analysis data support a positive association of risk with IGF1 serum levels [7,8]. In PCa cell culture models IGF or insulin stimulation or receptor overexpression triggered proliferation of malignant cells but enhanced differentiation in benign cells [9]. In a large recent tumor tissue microarray protein expression study including primary tumors of some 800 PCa patients the expression level of IGF1R was associated with a worse prognosis as was a decreased expression of PTEN [9]. Within the IGF network not only the IGF1R but also the INSR exerts cancer-promoting functions. This effect is driven by total insulin and insulin receptor (INSR) levels but also the ratio of the two INSR isoforms A and B (INSRA; INSRB) [10,11].

Taken together there is strong evidence for the IGF axis as a promising therapy target in PCa.

The IGF axis is a complex signaling network that is involved in many physiologic and oncologic processes, e.g. proliferation, survival, growth, energy provision and metabolism [12,13,14,15]. The whole IGF axis constitutes an interactive network composed of the peptide-ligands IGF1, IGF2 and insulin, and the receptors IGF1R, IGF2R and INSR as IGF binding proteins (IGFBPs) [16]. The IGF1R and the INSR receptor show a high degree of sequence and structural similarity allowing the formation of IGF1R/INSR hybrid receptors [17]. The INSR itself appears in two isoforms, INSRA and INSRB differing in 12 amino acids encoded by exon 11. By alternative splicing these amino acids are present in the C-terminal end of the insulin-binding beta subunit of INSRB but not in that of INSRA [18]. IGF1R, INSR and IGF1R/INSR hybrid receptors are activated by both IGFs and insulin ligands, which exhibit different affinities for the various receptor types. Receptor activation elicits downstream signals of the Ras-Raf-Erk and the PI3K/AKT/mTOR signaling pathways [6,19].

Preclinical results of IGF targeting therapies using antibodies blocking ligand binding to IGF1R or small molecule inhibitors of the receptors' kinase activity have shown promising responses and boosted clinical trials with these drugs. In addition, also indirect targeting strategies, for example by inhibition of PAPP-A, a protease increasing the bioavailability of IGFs through cleavage of IGFBP4 [6], or targeting of Lin28b, an RNA regulatory protein that stimulates genes of the IGF axis [20] or enhancement of inhibition by combined targeting with the small molecule receptor kinase inhibitor OSI-906 and siRNA knockdown of IGF1R have been suggested [21]. Currently more than 100 clinical studies examining a variety of anti-IGF1R agents in several cancer entities are ongoing [22]. However, the only and first phase III study with the IGF1R-targeting antibody figitumumab in combination with chemotherapy in non-small-cell lung cancer has been halted due to lack of efficacy as well as due to safety concerns such as hyperglycemia, hemorrhaging and hemoptysis, cardiovascular and cardiopulmonary failure [23]. A reason for the disappointing clinical results might be that fact that the complexity of the IGF axis has not been considered appropriately. The complexity and redundancy of the IGF network facilitate therapy escape mechanisms and its important physiological role controlling the cellular metabolism triggers side effects of IGF1R targeting therapies.

Recently, we reported that both stimulation of the IGF1R and INSRA by the ligands IGF or insulin have growth-promoting effects in PCa cells [24]. In the same line of evidence a kinome-wide screen in breast cancer identified the insulin and IGF pathway as an escape mechanism from hormone dependence in estrogen receptor positive breast cancers [25]. These findings favor a concept of co-targeting. In the present study we scrutinized the effect of INSRs in addition to IGF1R in PCa using *in vitro* and *in vivo* models. Briefly, we found that the INSRA drives oncogenic mechanisms equivalent to IGF1R and needs to be considered when designing clinical trials targeting the IGF axis. We further report differential functions of the IGF axis in cancer compared to non-cancerous prostate epithelial cells, a finding that might help to understand and avoid side effects of IGF targeting therapies.

## RESULTS

To investigate the functions of IGF1R and INSRs on cell proliferation, colony formation ability, cell migration, invasion and apoptosis, we overexpressed and downregulated IGF1R and INSR in cancerous and non-cancerous *in vitro* models of the prostate. The two isoforms of INSR (INSRA and INSRB) were described to exert differential functions [18,26,27]. Therefore we overexpressed INSRA and INSRB separately and selectively downregulated INSRB. Selective downregulation of INSRA was not possibly because of overlapping sequences between INSRA and INSRB (INSRA is lacking INSR exon 11). Successful target gene overexpression and downregulation using the described overexpression plasmids and siRNAs was previously confirmed by qPCR and Western Blot [28].

### IGF1R, INSR: effects on cell proliferation

We have previously shown that PCa cell lines respond to either IGF1R or INSRA overexpression with increased cell proliferation. INSRB overexpression did not influence the proliferative ability of the tested PCa cell lines. In contrast, the non-cancerous cell line EP156T responded to IGF1R and INSR overexpression with decreased cell proliferation and enhanced differentiation [29].

**Figure 1:**
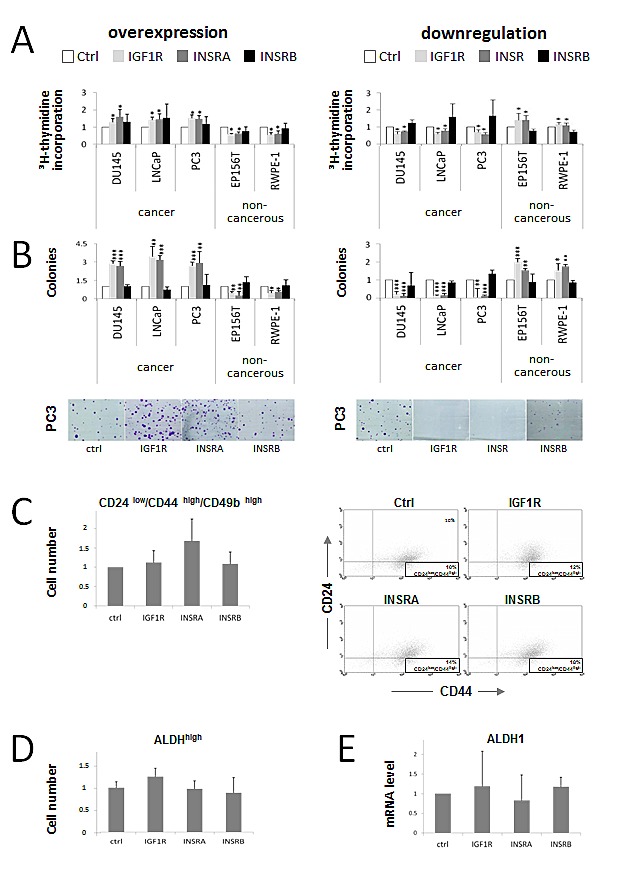
IGF1R/INSRA expression levels influence PCa cell proliferation and colony formation potential but have minor effects on cancer stem/progenitor cell marker levels A) IGF1R/INSRA impact on PCa cell proliferation. New DNA synthesis determined by thymidine incorporation assay was measured to assess cell proliferation in PCa cells (DU145, DuCaP, LNCaP and PC3) and non-cancerous prostate cells (EP156T and RWPE-1) following IGF1R, INSRA or INSRB overexpression using overexpression plasmids as well as IGF1R, INSR or INSRB downregulation applying specific siRNAs. B) IGF1R/INSRA modulate the colony formation potential of PCa cells. Relative number of colonies of PCa cells (DU145, DuCaP, LNCaP and PC3) and non-cancerous prostate cells (EP156T and RWPE-1) following IGF1R/INSR overexpression and downregulation was determined by 2D colony formation assay. Not only colony sizes, but also colony numbers were strongly influenced by cellular IGF1R/INSR expression levels. C) Identification of the cancer stem/progenitor cell marker panel CD24^low^/CD44^high^/CD49b^high^ in PCa cells overexpressing IGF1R, INSRA and INSRB (data shown for PC3). Cells transfected with IGF1R/INSRA/INSRB overexpression plasmids were analyzed for CD24, CD44 and CD49b expression and compared to cells transfected with ctrl plasmid. On the right representative dot blots of CD49b positiv control cells and cells overexpressing IGF1R, INSRA and INSRB, respectively, analyzed for CD44 and CD24 expression are shown. D) ALDH activity in PCa cells overexpressing IGF1R/INSR (data shown for PC3 cells). ALDH activity was analyzed by flow cytometry and compared to control cells. A specific ALDH inhibitor (DEAB) was used as a control for each sample to define and subtract background fluorescence. E) ALDH1 mRNA levels in PCa cells following IGF1R/INSR overexpression (data shown for PC3 cells). Data are presented as mean ± SD of a minimum of four independent experiments. Statistics, Student's t-test.

Here we confirmed these data using an alternative assay for proliferation, the thymidine incorporation assay, which measures new DNA synthesis instead of total cell numbers: Overexpression of the IGF1R and INSRA increased cell proliferation in PCa cell lines and decreased cell proliferation in non-cancer cell lines (Fig 1A). Vice versa downregulation of either IGF1R or total INSR decreased cancer cell proliferation while increasing proliferation in non-cancerous cell lines (Fig 1A). In both, the overexpression and downregulation studies selective regulation of INSRB did not influence cell proliferation of either cancerous or non-cancerous prostate cells (Fig 1A). Taken together we confirm here our previous data that IGF1R and INSRA mediate proliferative signals in PCa cells while enhancing differentiation accompanied by decreased cell growth in non-cancerous prostate cells.

### IGF1R, INSR: effects on colony formation ability

We now extended our study and investigated the impact of IGF1R and INSR level modulations on colony formation ability, an additional cancer-promoting feature. To monitor the colony formation ability of PCa and non-cancerous prostate cells the cells were transfected with overexpression plasmids or siRNA prior seeding them at low density in culture flasks. We intended to place a main focus on the initial phases of colony establishment, i.e. single cell survival, adhesion and colony initiation, rather than on proliferative features. Therefore no further transfections were performed during the incubation time of the colony formation assay (10-14 days). Overexpression of IGF1R and INSRA increased colony formation of cancer cells (Fig 1B). Not only colony size but also colony numbers increased markedly indicating that overexpression of IGF1R and INSRA did not only promote proliferation of PCa cells but also single cell survival and colony establishment (Fig 1B). We wondered whether enhanced “stemness” properties of PCa cells caused by IGF1R/INSR overexpression are responsible for the observed phenotype. Therefore we analyzed known cancer stem cell markers. The expression pattern of the cancer progenitor/stem cell-like marker panel (CD24^low^/CD44^high^/CD49b^high^) remained unchanged in PCa cells overexpressing IGF1R or INSRs. Overexpression of INSRA, however, resulted in a moderately increased amount of cells with cancer progenitor/stem cell-like features (Fig 1C). High ALDH activity, characteristic for tumor initiating cells, as well as ALDH1 mRNA levels remained unaltered upon IGF1R/INSR receptor overexpression (Fig 1D). Apart of a moderate increase of CD24^low^/CD44^high^/CD49b^high^ cells upon INSRA overexpression, no enhanced progenitor/stem cell-like phenotype was observed in IGF1R/INSR overexpressing cells indicating that enhanced “stemness” properties are possibly not responsible for the increased number of colonies upon IGF1R/INSR overexpression.

IGF1R and INSR downregulation reduced colony formation potential of PCa cells almost completely (Fig 1B). Modulations of only INSRB had no major impact on colony formation ability of cancer cells suggesting that INSRA is the major INSR isoform promoting colony formation ability (Fig 1B). Interestingly, in non-cancerous cells the effects were reversed: downregulation of IGF1R and INSR increased colony formation ability while IGF1R/INSR overexpression decreased colony formation ability (Fig 1B). We have previously shown that IGF1R and INSR overexpression promotes basal to luminal differentiation in non-cancerous prostate cells [30]. Basal cells feature a higher colony formation capacity compared to luminal cells. Thus induced differentiation upon IGF1R/INSR overexpression supposable mediates decreased colony formation ability in non-cancerous prostate cells.

### IGF1R/ INSR effects on cell migration and invasion

The ability of cancer cells to migrate and invade to extracellular matrix is an important feature for cancer progression and metastasis formation in all types of cancer [31,32]. Thus we investigated the effect of IGF1R and INSR overexpression and downregulation on cell migration and invasion using a Boyden chamber assay.

**Figure 2:**
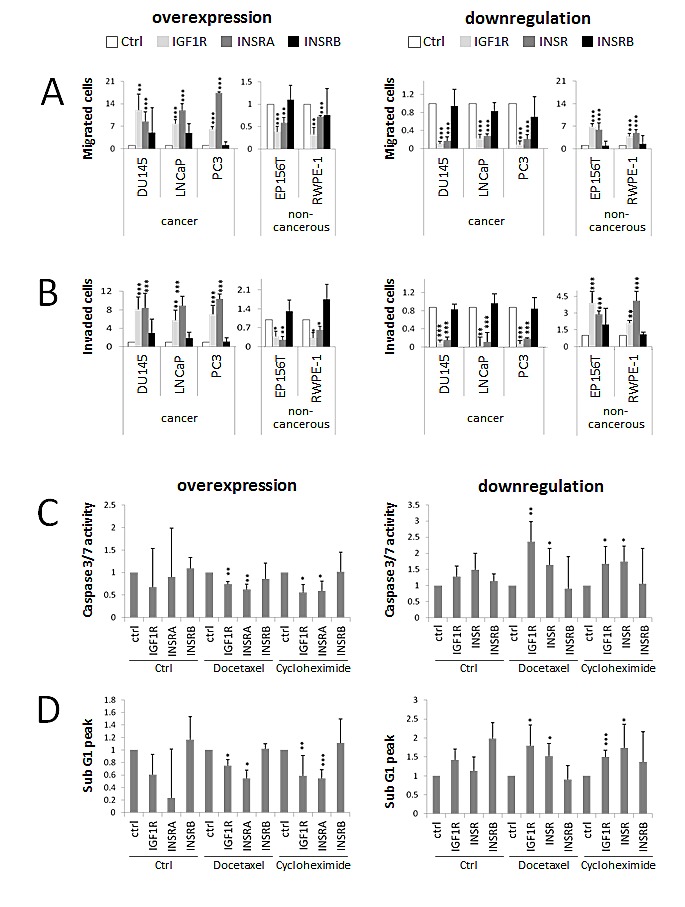
IGF1R/INSRA expression levels influence PCa cell migration, invasion and resistance to apoptosis A) IGF1R/INSA influence PCa cell migration. Migrated PCa (DU145, DuCaP, LNCaP and PC3) and non-cancerous prostate cells (EP156T and RWPE-1) normalized to total cell numbers were analyzed in a Boyden chamber assay following IGF1R/INSR overexpression or downregulation using overexpression plasmids (IGF1R, INSRA, INSRB) and specific siRNAs (IGF1R, INSR, INSRB), respectively. B) IGF1R/INSA influence PCa cell invasion. Invaded cells were analyzed in a Matrigel-coated Boyden chamber assay and normalized to total cell numbers. C-D) IGF1R/INSRA levels impact on PCa sensitivity to apoptotic stimuli such as treatment with docetaxel or cylcohexamide. PCa cells (PC3 cells shown) were transfected with overexpression plasmids or siRNAs specific for IGF1R/INSR prior exposure to apoptotic stimuli. Apoptosis was measured by determining caspase 3/7 activity (Caspase activity ELISA, C) or propidium iodide staining (flow cytometry analysis, determination of sub-G1 fraction, D). Data are presented as mean ± SD of a minimum of four independent experiments. Statistics, Student's t-test.

Boyden chambers were used as un-coated (migration) or matrigel-coated inserts (invasion). Migrated/invaded cells were normalized to total cell numbers in order to reduce the impact of proliferative effects. IGF1R and INSRA overexpression increased the migration (Fig 2A) and invasion (Fig 2B) potential of all the tested cancer cell lines, while IGF1R and INSR downregulation reduced cell migration (Fig 2A) and invasion (Fig 2B). Selective overexpression or downregulation of INSRB had no major impact on cell migration and invasion (Fig 2A and 2B). Again, non-cancerous cells responded to changes in IGF1R and INSRs levels conversely to cancer cells: IGF1R/INSRA overexpression caused decreased migration (Fig 2A) and invasion (Fig 2B), while IGF1R/INSR downregulation was associated with enhanced migration (Fig 2A) and invasion (Fig 2B).

### IGF1R/INSRs effects on cell survival following apoptotic stimuli

IGF signaling is known to enhance pro-survival pathways such as the PI3K-AKT pathway [33,34]. Next we investigated the influence of IGF1R and INSR on cells, which were exposed to apoptosis-inducing agents. IGF1R and INSR were overexpressed or downregulated for 48 hours prior induction of apoptosis by docetaxel (chemotherapeutical drug for PCa treatment) or cycloheximide (translation inhibitor, positive control). We confirmed induction of apoptosis by docetaxel and cycloheximide applying two independent apoptotic measurements: propidium iodide staining (FACS analysis, sub G1 peak) and caspase 3/7 activity (Caspase activity ELISA). Overexpression of IGF1R and INSRA but not INSRB decreased apoptosis behavior in PCa cells, however downregulation of IGF1R and INSR (not INSRB) increased apoptosis in PCa cells in both apoptosis assays (shown for PC3) (Figure 2C-2D). For benign cells no change in apoptosis behavior has been observed upon overexpression or downregulation of IGF1R and INSR (data not shown). These data indicate that, under selection pressure such as chemotherapeutic cancer treatment, overexpression of IGF1R and INSR prevents PCa cells from apoptosis. Conversely, IGF1R/INSR downregulation makes PCa cells more sensitive to chemotherapeutic treatment.

### IGF1R/INSR effects on tumor growth and angiogenesis *in vivo*

**Figure 3:**
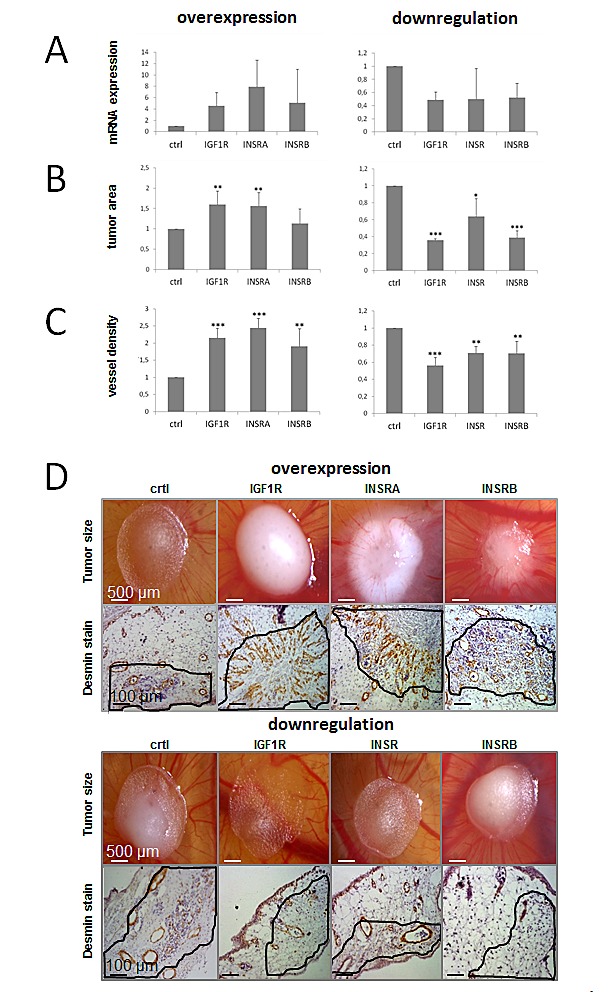
IGF1R/INSR expression levels influence tumor growth and tumor-infiltrating blood vessel density *in vivo.* Data were achieved using the chicken chorioallontoic membrane (CAM) assay and the PCa model system PC3. A) Successful target overexpression (IGF1R, INSRA, INSRB) or downregulation (IGF1R, INSR, INSRB) was confirmed by qPCR after harvesting of the onplant tumors B) IGF1R/INSR expression levels influence tumor size in vivo. C) Tumor IGF1R/INSR levels impact on the amount of tumor-infiltrating vessels determined by visualization of desmin-immunoreactive pericytes by immunohistochemistry. D) Representative pictures of tumor onplants and anti-desmin staining to visualize tumor-infiltrating blood vessels. Data are presented as mean ± SD of three independent experiments analyzing four different onplants per CAM and treatment. Statistics, Student's t-test.

In the past three decades, the chorioallontoic membrane (CAM) assay has been developed to an accepted and reliable *in vivo* model for testing substances or to monitor angiogenic processes and tumor growth [35]. PC3 cells have been previously established in a CAM assay [36], so we investigated the effects of changes in IGF1R/INSR levels on tumor growth and vessel density using PC3 PCa cells. Transfections for transient IGF1R/INSR overexpression or downregulation were performed once prior placing the tumor cells on the CAM. Successful target overexpression or downregulation was verified by qPCR after harvesting the onplants (Figure 3A). No further transfections were performed. In line with the *in vitro* data overexpression of IGF1R and INSRA but not INSRB significantly increased tumor area, while downregulation of the IGF1R and the INSR strongly reduced tumor growth in the CAM-assay (Figure 3B-3D). In contrast to the *in vitro* results, also downregulation of the INSRB resulted in a decreased tumor area. Apart of tumor growth we also investigated angiogenesis in this model by determining vessel density and staining of desmin-immunoreactive pericytes (Figure 3C-3D). We report here, firstly, that not only IGF1R but also INSR promotes angiogenesis *in vivo*. Interestingly, in this assay both isoforms of the INSR had comparable effects.

## DISCUSSION

The dysregulation of the IGF axis and its distinct oncogenic driver function in several cancer entities [10,37,338,^39^] makes the IGF axis an attractive therapeutic cancer target. Currently more than 100 clinical trials are evaluating the effect of IGF1R targeting therapeutics as single agents or in combination with standard treatments in several cancer entities [40]. Promising results from *in vitro* and mouse studies have encouraged clinical studies with different IGF1R monoclonal antibodies or tyrosine-kinase inhibitors. However, the outcomes so far do not come up to the expectations. Lack of efficacy and several side effects such as hyperglycemia led to a premature discontinuation of the phase III IGF1R targeting therapy study in non-small cell lung cancer. A better understanding of the function(s) of the complex IGF network in malignant and non-malignant tissues is in demand to improve these therapy approaches and interpret the outcomes.

This study was undertaken to investigate the role of IGF1R and INSRs in PCa and to compare the impact of the IGF axis in PCa and non-cancerous prostate epithelium. Employing *in vitro* and *in vivo* models we investigated the effect of IGF1R and INSRs level changes on several oncogenic hallmarks and found that the IGF1R and the INSRA both act as oncogenes. The present data clearly show that overexpression of either of the two receptors increases tumor growth, proliferation, colony formation, migration, invasion and angiogenesis *in vitro* and *in vivo* in PCa. Vice-versa a downregulation of the IGF1R and the INSR but not the INSRB alone strongly inhibited the PCa cells. These findings identify INSRA in addition to IGF1R as a prostate tumor driver and a promising therapy target. In contrast, INSRB had no impact on cancer cell proliferation, colony formation, migration and invasion potential *in vitr*o suggesting a differential function of the INSR isoforms on PCa cells. This might be achieved by the higher affinity of INSRA for IGF growth factors or differences at the C-terminal end of the insulin-binding alpha subunit potentially eliciting different receptor downstream signals [41]. Blood vessel formation seems to be an exception of the different role of INSRB. Its overexpression stimulated blood-vessel formation in the CAM model, although less than IGF1R and INSRA. A possible explanation for this finding might be a different ligand spectrum and different ligand concentrations in the CAM *in vivo* model compared to cell culture conditions or distinct signals promoting angiogenesis and other cancer-promoting features.

The INSR receptor isoform A has been mainly associated with proliferative effects and was found overexpressed or overexpressed in relation to INSR isoform B in cancer [10,42,43]. INSR isoform B, on the other hand seems to be predominantly serving a metabolic function. The fact that IGF1R and INSRA both bind IGFs with high affinity might be the joint mechanism to growth-driving effects of these two IGF-axis receptors [44]. Taken together these results support therapeutic targeting approaches directed towards both, IGFR1 and INSRA for efficient antitumor activity and sparing INSRB to minimize side effects.

Major problems of conventional cancer therapy by chemotherapeutic agents are unsatisfying therapy efficacy on one hand and tumor relapse after a certain treatment time on the other hand. We found that IGF1R/INSRA overexpression makes cells less sensitive to induction of apoptosis, while IGF1R/INSR downregulation enhanced the induction of apoptosis following treatment with chemotherapeutic agents such as Docetaxel. These data indicate that combination therapies of anti-IGF1R (and/or anti-INSRA) with standard chemotherapeutic agents may not only profit from the combined effect of the single therapies but also from the fact, that an anti-IGF1R (and/or anti-INSRA) therapy makes cancer cells more sensitive to chemotherapeutic/apoptotic agents.

Tumor blood vessel formation is a pivotal step of carcinogenesis and metastasis. Our results on stimulation of *in vivo* angiogenesis by IGFR1/INSR overexpression demonstrate an important role of the IGF axis in this process. This is in agreement with reports on stimulation of neo-angiogenesis in wound healing and tumor angiogenesis by insulin and IGF1 and its attenuation by the growth factor sequestrating IGF-binding protein 3 [45,46]. In gastric and endometrial cancer models the angiogenesis promoting effect of insulin/IGF signaling was reported to be mediated by VEGF [47,48,49].

Considering the fact that the IGF axis has many important physiological functions we additionally investigated the role of the receptors in non-cancerous prostate epithelial cells (EP156T, RWPE-1). In contrast to tumor cells non-cancerous cells exhibited converse effects in response to receptor up- and downregulation. This is in line with our previous finding that non-cancerous cells undergo differentiation in response to stimulation of their IGF axis rather than proliferation [50]. Obviously, receptor activation triggers divergent downstream pathway(s) and biological effects in malignant and non-malignant cells.

## CONCLUSION

In conclusion the present study provides evidence that IGFR1 and INSR isoform A are both strong oncogenic drivers in PCa. Consequently, co-targeting of INSRA and IGF1R may be a promising approach to enhance the efficacy of anti-IGF-receptor therapies, possibly without affecting the physiological functions of INSR, which can still be mediated by INSRB. Moreover new insights into the biology of the prostate and prostate cancer are provided, indicating that the IGF axis exerts different functions in malignant and non-malignant cells, a finding that might help to better understand and avoid some side effects of IGF1R targeting agents.

## MATERIALS AND METHODS

### Cell lines and Cell culture

The PCa cell lines PC3, DU145 and LNCaP represent derivatives of PCa metastases and were obtained from the American Type Culture Collection (ATCC). EP156T and RWPE-1 represent immortalized derivatives of benign prostate epithelial cells and were established by overexpression of hTERT and HPV18, respectively [51,52]. The identity of the used cancer cell lines was confirmed by forensic DNA fingerprinting methods using the AmpFlSTR^®^ SGM Plus^®^ PCR amplification kit (Applied Biosystems). Cell lines were cultured in growth media with supplements as previously described [53,54,55].

### Transient overexpression

150,000 (PC3, DU145, LNCaP) or 200,000 (EP156T, RWPE-1) cells per well were seeded into 6 well plates. The next day the cells were transfected with the following expression plasmids: IGF1R (pRK5), INSRA (pRcCMVi), INSRB (pRcCMVi), green fluorescent protein (GFP, pMaxGFP); or empty control vector (Flag tagged pRcCMV) [56]. PC3, DU145 and LNCaP were transfected using Nanofectin transfection reagent (PAA laboratories), while EP156T and RWPE-1 were transfected using Fugene HD transfection reagent (Roche) following the manufacturer´s instructions. 48 hours after transfection the cells were harvested. Experiments were performed in full growth medium in the presence of FCS (cancer cells) or FCS, pituitary extract and supplemented growth factors (non-cancerous cells). Transfection efficiencies were analyzed using pMaxGFP plasmid and flow cytometry analysis. Transfection efficiencies varied from 87% to 96% as previously described [57]. Target gene overexpression has been previously confirmed by qPCR and Western blot [58].

### siRNA knockdown

150,000 (PC3, DU145, LNCaP) or 200,000 (EP156T, RWPE-1) cells per well were seeded into 6 well plates. Transfection of receptor-targeting or control siRNAs was performed the following day using Nanofectin short interfering RNA (siRNA) transfection reagent (PAA laboratories) according to the manufacturer´s instruction. IGF1R and INSR siRNAs were designed and synthesized by Invitrogen (siIGF1R: sense 5'-CAACAGUGGUCAUCAUGGAACUGAUdTdT-3', siINSR: sense 5'-UAGCUGAGCUGCCAGAUUGUUGCCUdTdT-3'). Control siRNA was obtained from Dharmacon (siControl: ON-TARGETplus Non-Targeting Pool). A specific siRNA for INSRA could not be designed because INSRA is identical to INSRB mRNA except for 36 base pairs (exon 11) present in INSRB only [59]. As reported previously, we designed an INSRB-specific siRNA within these 36 base pairs (siINSRB sense 5'-ACUGGUGCCGAGGACCCUAtt-3') [60]. Downregulation of INSRB using specific siRNA was confirmed by qPCR as previously described [61]. All experiments were performed in full growth medium. 72 hours after transfection the best target gene downregulation was obtained. Efficient target gene downregulation was confirmed by qPCR and Western blot analysis (see [62]).

### Quantitative real-time PCR (qPCR)

Total RNA was isolated using the RNeasy mini kit (Qiagen). cDNA synthesis was performed using iScript select cDNA synthesis kit (Bio-Rad Laboratories). qPCR was performed on an ABI Prism 7500 fast real-time PCR System (Applied Biosystems, Life Technologies) as previously described [63,64]. Primer and TaqMan probes for IGF1R, INSR, INSRA, INSRB and the endogenous control HPRT1 were designed according to sequences from the Nucleotide Sequence Database NCBI using ABI Prism Primer Express Software 2.0.0 (Applied Biosystems, Life Technologies): IGF1R (forward primer, 5'-CCTCCAACTTCGTCTTTGCAA-3'; reverse primer, 5'-CAGGTCACTGGCCCAGGA-3'; probe, 5'-TGCCCGCAGAAGGAGCAGATGACA-3'); INSR (forward primer, 5´-CAAGTGCATCCCTGAGTGTCC-3´; reverse primer, 5´-CGAGTCGATGGTCTTCTCGC-3´; probe, 5'-GATGAATTCCAGCAACTTGCTGTGACC-3'); INSRA (forward primer, 5´- tggttttcgtccccaggcc-3´; reverse primer, 5´- ccaccgtcacattcccaac-3´; probe, 5'-tctcggaaacgcaggtcccttggcga-3'); INSRB (forward primer, 5´-gtgccgaggaccctaggcc-3´, reverse primer, 5´-ccaccgtcacattcccaac-3´, probe, 5'-tctcggaaacgcaggtcccttggcga-3') and HPRT1 (forward primer, 5´-gctttccttggtcaggcagta-3´; reverse primer; 5´- gtctggcttatatccaacacttcgt-3´; probe, 5'-gtctggcttatatccaacacttcgt-3'). The two isoforms of the INSR (INSRA and INSRB) differ from each other in a 36 base pair exon (exon 11) present in INSRB, but not in INSRA [65]. We developed two TaqMan gene expression assays differentiating between INSRA and INSRB by designing selective forward primers crossing the exon-exon boundary exon 10-exon 11 (INSRB) or exon 10-exon 12 (INSRA) and identical reverse primers and probes. All TaqMan probes were labeled with 6-Fam reporter dye and Tamra quencher dye. TaqMan gene expression assays were performed as previously described [66,67].

### [^3^H]Thymidine incorporation assay

Cells were seeded in triplicates onto 96-well plates. On the next day, the cells were transfected with overexpression plasmids or siRNA as described above. 25 μL/well of [^3^H]thymidine (1 μCi/well) were added to cells. The day after DNA was harvested on 96-well filter plates (UniFilter; Perkin-Elmer), Scintillation fluid (50 μL) was added and radioactivity was quantified using Chameleon 5025 liquid scintillation counter (HVD Life Sciences).

### Clonogenic assay

48 hours after transfection cells were washed in PBS, trypsinized, harvested and total cell number of viable cells were determined using CASY cell counter and analyzer system (Schärfe System). Afterwards 1,000 cells were seeded into a 75 cm^2^ cell culture flask with 12 ml cell specific medium and incubated for 10 days with a medium change at the fifth day. After 10 to 14 days cells were fixed with 100% ice-cold Methanol for 5 minutes and stained with a 0.5% cystal violet solution (Sigma Aldrich) for 2 minutes. Colony numbers were determined with a CCD camera with green electro luminescent transillumination.

### Caspase activity assay

For the measurement of the caspase-3/7 activity, cells were transfected with overexpression plasmids or siRNA for 48 hours or 72hours, respectively. Cells were harvested and cell pellets were lysed in 50 μl assay lysis buffer. Afterwards Caspase-Glo assay (Promega) was performed according to the manufacturer's instructions. Caspase 3/7 activity was quantified using Chameleon 5025 counter (HVD Life Sciences).

### Flow cytometry

Cells were seeded in 6-well plates and transfected with overexpression plasmids or siRNA as described above. Afterwards cells were trypsinized and cell pellets were suspended in propidium iodide (PI) buffer (0.2% Triton-X-100, 2 ng/mL Na-Citrate, and 0.1 mg/mL PI) and kept light-protected at 4°C for 1 hour.

Apoptosis (subG1 fraction) was analyzed using FACS Calibur (Becton Dickinson). For analysis of the stem cell-like phenotype cells were detached by scraping, washed with PBS containing 2% BSA, stained with anti-CD24-FITC, anti-CD44-PerCP-Cyc5.5 and anti-CD49b-PE (all from BD) and incubated at 4°C for 30 minutes. After incubation, cells were washed once with PBS containing 2% BSA and the CD24^low^/CD44^high^/CD49b^high^ population was determined using FACS Calibur.

### ALDEFLUOR assay

ALDEFLUOR detection kit (StemCell Technologies) was used to identify cells that exhibit high activity levels of the stem cell marker ALDH (aldehyde dehydrogenase). ALDH substrate was added to the cells, converted to a fluorescence product in the presence of active ALDH and analyzed by flow cytometry. As control for background fluorescence a specific inhibitor of ALDH, dieethylaminobenzaldehyde (DEAB) was used according to manufacturer´s instructions.

### Migration/Invasion assay

Following transfection with siRNAs or plasmids cells were seeded on the upper sides of boyden chambers (24-well BD cell culture inserts with 8 μM membrane pore size (Becton Dickinson). Media with 1 % FBS in the upper chamber and 10 % FBS in the lower chamber were used to form a gradient to induce cell migration. For invasion assay the inserts were matrigel coated 24 hours prior to seeding the cells. 48 hours after seeding the cells in the upper chamber were removed with cotton swamps and the cells, which had migrated/invaded to the lower chamber were fixed with ice-cold methanol and stained with nuclear stain DAPI. The fixed cells were visualized with a fluorescence microscope (Zeiss, Axiovert) and quantifified (TissueFax, TissueGnostics). The numbers of migrated/invaded cells were normalized to total cell numbers estimated by EZ4U cell viability assay (Bionet).

### Preparation of onplant tumor xenografts for the chorioallantoic membrane (CAM) model

Tumor onplants were prepared as previously described [69]. Briefly, native, non-pepsinized, type I rat tail collagen (BD Bioscience) was neutralized with 0.1 M NaOH and mixed with 10x DMEM medium (Gibco) on ice. 300,000 PC3 cells, previously transfected with siRNA (siControl, siIGF1R, siINSR, siINSRB) or overexpression plasmids (empty control, IGF1R, INSRA or INSRB), were added to 30 μL collagen solution, and dropped into a petri dish covered with a sterile parafilm. Collagen drops were coagulated at 37°C for 45 minutes and applied to the CAM.

### Ex-ovo chick chorioallantoic membrane (CAM) assay

The CAM-assay was performed as described elsewhere [69] with slight modifications. In brief, fertilized white leghorn chicken eggs (SPF eggs) were purchased from Charles River (Germany) and incubated at 37° C with 80% humidity (Grumbach BSS 160 MPGTFS) for three days. At day three, eggs were opened and transferred to plastic weighing boats. *Ex ovo* cultures were covered with a square petri dish and placed into a stationary incubator at 37°C and 80% humidity for six days. Then, collagen-onplants with PC3 cells were applied to CAMs (four equal onplants/CAM) and incubated for five days. Xenografts were analyzed under a stereomicroscope with a connected digital camera and flexible cold light (Olympus SZX10, Olympus E410). For histological analysis onplants were excised from the CAM, fixed in 4% paraformaldehyde solution and processed for paraffin sectioning. To determine the tumor area the ImageJ program (NIH,USA) was used. Successful target gene overexpression or downregulation was analyzed by qPCR as described above.

### Immunohistochemistry

Tissue sections from CAM xenograft onplants were deparaffinizedand de-hydrated in graded alcohol series and xylene. Antigen retrieval was performed in a water bath (95°C) for 20 minutes using target retrieval solution (Dako Cytomation). Endogenous peroxidase activity was blocked with 3 % H_2_O_2_/methanol. Serial sections were incubated in blocking solution containing 10 % fetal calf serum (Dako Cytomation) for 45 minutes, stained for one hours with monoclonal mouse anti-Desmin antibody (clone CD33, Dako Cytomation) followed by a biotinylated secondary antibody (biotinylated rabbit anti-mouse IgG, Vector Laboratories Inc.), and visualized using the Vectastain Elite ABC Kit (Vector Laboratories Inc.) and the FAST DAB Tablet Set (Sigma Biochemicals). Sections were counterstained with Meyer's hematoxylin, mounted with Pertex (Medite) and analyzed on an inverse microscope (Zeiss Axiovert 200M with Axiovision 4.7 Software, Carl Zeiss Optics). For blood vessel calculations, sections from the central region of the xenograft onplants were selected (four different xenografts for each treatment).

### Statistical analyses

Student's t-test was applied for calculating the statistical significance of differences between the treatment groups. P-values below 0.05 were considered significant (* P<0.05; ** P<0.01; *** P<0.001). Bars and error bars in the histograms represent mean values ± standard deviation (SD) of at least four independent experiments.

## Abbreviations

ATCC: American type culture collection
CAM: Chorioallantoic membrane
FCS: Fetal calf serum
IGF: Insulin-like growth factor
IGF1R: Insulin-like growth factor receptor 1
INSR: Insulin receptor
INSRA: Insulin receptor isoform A
INSRB: Insulin receptor isoform B
siRNA: small interfering RNA
PCa: Prostate cancer

PM is currently working in a DAAD fellowship at Roche Diagnostics GmbH. The work presented here is independent on the fellowship or any work related to Roche Diagnostics GmbH.
